# A Case Report of Leukocytosis During Modified Electroconvulsive Therapy of Paranoid Personality Disorder

**DOI:** 10.3389/fpsyt.2022.899847

**Published:** 2022-05-26

**Authors:** Xingyu Mu, Jiao Xu, Peilin Lin, Ya Luo, Yuan Zhu, Yi Shi, Shangtao Chen, Zengxiang Wu, Shuangqing Li

**Affiliations:** ^1^Department of General Practice, West China Hospital, Sichuan University, Chengdu, China; ^2^The University of Edinburgh, Edinburgh, United Kingdom; ^3^Mental Health Center, West China Hospital of Sichuan University, Chengdu, China

**Keywords:** paranoid personality disorder, modified electroconvulsive therapy (MECT), leukocytosis, neutrophils, monocytes, case report

## Abstract

**Introduction:**

Modified electroconvulsive therapy (MECT) is a viable therapeutic option for patients with mood disorders and schizophrenia. We found that there is a relationship between MECT and leukocytosis. To the best of our knowledge, this is the first case of this problem. There are no relevant guidelines recommending the risk of leukocytosis caused by MECT, nor the method to reduce the risk. We hope to share this case to provide a reference for the prevention and treatment of similar patients with leukocytosis during or after MECT and remind psychiatrists to pay attention to this risk of leukocytosis before making the decision of MECT while knowing how to deal with it.

**Case presentation:**

We describe a case of a 24-year-old woman diagnosed with Paranoid personality disorder (PPD) whose symptoms began at 19 years old. Her main clinical manifestations are feeling targeted, cheated, tracked, misunderstood, and repeating action. Since antipsychotic treatment was ineffective, we considered MECT. After MECT, the patient’s body temperature increased, and leukocytosis was found. After excluding infection and other possibilities, we added 1,000 ml physiological saline to the patient through the vein. The white blood cell (WBC) count returned to normal in a short time.

**Conclusion:**

Before MECT, it is necessary to screen blood cytology. During and after MECT, we should be alert to leukocytosis that may be related to MECT and deal with it correctly in time.

## Introduction

Paranoid personality disorder (PPD) is a mental disorder that has always been neglected clinically, but it is indeed a serious and difficult problem to treat; it is often manifested as suspicion, excessive attention to oneself, hostility, etc. (1). The patient was diagnosed with PPD after admission and received medication including clozapine, risperidone and benzhexol hydrochloride tablets. The patient’s symptoms did not improve after treatment, so we added MECT as well as continuing medical treatment. MECT, also known as non-convulsive (NC)-ECT, is considered an important physical therapy for mental diseases with the advantages of safety, quick-acting and efficiency (2). MECT is widely used in the clinic and has become an indispensable psychiatric treatment and has advantages for treatment-resistant schizophrenia and recurrent refractory mania (3). Previous studies have shown that continuous electric convulsive treatment (ECT) is safe, effective and low cost (4). However, studies have also suggested that MECT may induce a change in the diversity of neurobiochemists, such as changes in interleukin (IL)-6, IL-1, IL-5, and CRP in depressed patients (5). It can also induce a change in the diversity of neurobiochemists (6). In this case report, we found that the patient had transient leukocytosis after receiving MECT, which has not been reported. Therefore, we discussed the causes of leukocytosis in combination with the complete treatment process. We hope that through this case report, clinicians, especially psychiatrists, can be alert to leukocytosis caused by MECT and know the existence of this risk and understand the most basic treatment when leukocytosis occurs.

## Presentation of the Case

A 24-year-old woman presented to the West China Hospital of Sichuan University with a 5-year history of auditory hallucination and feeling of being revealed and had no history of allergies or special physical diseases. Nine years ago (January 21, 2013), the patient had no obvious reason to feel targeted, cheated, tracked, misunderstood, repeatedly checked whether the door was locked, and even did not believe in family, declined academic performance, was hospitalized in West China Hospital of Sichuan University and was diagnosed with “delusional disorder”. When hospitalized, the patient had passive contact and was not friendly to the medical staff and her families, which can lead to relationship delusion, lack of insight, and serious damage to social function. After admission, EEG and MRI were normal, and psychiatrists administered risperidone, quetiapine fumarate, clonazepam, benzhexol hydrochloride and escitalopram oxalate. The patient was discharged from the hospital after the symptoms were relieved, but soon after discharge, the patient stopped taking medicine without the guidance of a doctor and did not meet the psychiatrists regularly.

Soon after, the patient dropped out of school, constantly changing her jobs, and the feeling of being revealed existed for a long time. Four years ago, the patient’s symptoms worsened and became more complicated. She became lazy, irritable, refused to go out, and sometimes smashed belongings, was hospitalized again on August 2, 2018, and was diagnosed with “schizophrenia.”

Her vital signs were normal, and she had normal blood pressure while seated (104/71 mmHg). The skin, head, neck, heart, lungs, abdomen and limbs were normal, with clear consciousness and natural expression, neat and timely clothes, concentration, answer questions to the point, have normal computing ability (we adopted the method of 100-7-7), and have good long-term memory and short-term memory (we asked the name of the teacher in junior high school and the catering content of last night, and the family members judged whether it was correct). The mechanical memory and logical memory were normal (we tested the mechanical memory by reciting five numbers forward and four numbers backward; we tested the patient’s logical memory by whether the patient could describe her condition in detail, which was also judged by the patient’s family members). The patient’s ability to orient to time, space and people is normal. We failed to lead to anxiety, depression, high mood, contradictory mood, relationship delusion and obsessive-compulsive disorder, but we led to the sense of being monitored, discussed and revealed, and the patient’s insight was partially impaired.

**FIGURE 1:**
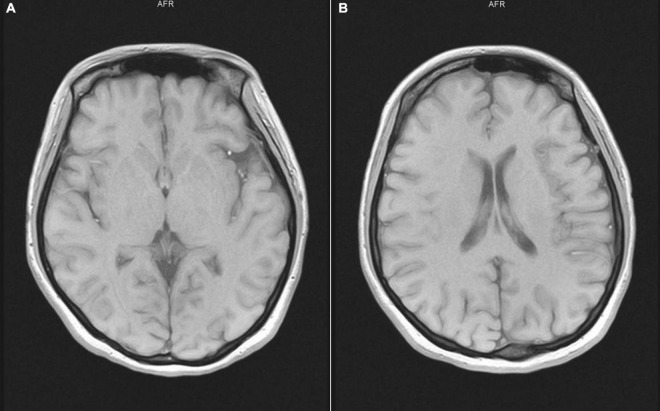
Cranial magnetic resonance scan showed microischemia on the bilateral frontal lobe. **(A,B)** show that different sections of the patient’s head MRI are normal.

Laboratory tests showed that the patient’s blood cell count, coagulation, kidney, liver and thyroid functions were normal, and the electrocardiogram (ECG) and electroencephalogram (EEG) were normal. A cranial magnetic resonance scan showed microischemia on the bilateral frontal lobe (Figure 1).

After reviewing the patient’s medical history and medication history, combined with the current situation, we decided to use clozapine 25 mg once in the morning, 50 mg once in the evening, risperidone 2 mg twice a day and benzhexol hydrochloride tablets 2 mg twice a day for treatment. We gradually increased the doses of clozapine and risperidone according to the patient’s situation within 14 days, and finally, the doses of clozapine and risperidone reached 150 and 6 mg/d, respectively. During this period, propranolol hydrochloride was used for a short time to slow down the patient’s tachycardia due to medication, and a topiramate capsule was used for 1 day before convulsion-free electroconvulsive treatment, but the patient’s condition did not improve.

Due to the persistence of delusion, we decided to try MECT. We fully communicated with the patient and guardians about the benefits and risks of MECT and obtained the consent of the patient and guardians. On August 15, 2018, before the patient received MECT for the first time, we conducted regular laboratory examination during hospitalization, which showed that the patient’s blood cell count, liver function and renal function were normal. Before MECT, we once again monitored the vital signs of the patient, body temperature: 36.3 degrees Celsius, pulse: 78 times/min, blood pressure: 110/66 mmHg, and confirmed that the patient had no discomfort. We used the American THYMATRON electric spasm therapeutic instrument, the model is system IV, which is an automatic electric spasm therapeutic instrument, the internal output voltage is 450 V and the output current is 900 mA. When starting the treatment, the energizing energy was 20% J, because of unstable current during the treatment process, the instrument automatically adjusted the current to 920 mA, and the energizing time was 5.6 s. The treatment process was successful. During and after MECT, the patient had very slight and unsustainable limb vibration lasted nearly 10 min, with no other symptoms. Vital signs remained stable, which was relieved after several minutes, and she was escorted back to the ward by guardians and doctors. However, after returning to the ward, the patient developed a fever, with a maximum temperature of 38.2 degrees Celsius. After we gave a short physical cooling, the patient’s temperature returned to normal, but the doctor on duty did not arrange the relevant laboratory examination on that day. The reason for the patient’s limb vibration and fever after MECT on the first day lacked evidence.

**FIGURE 2:**
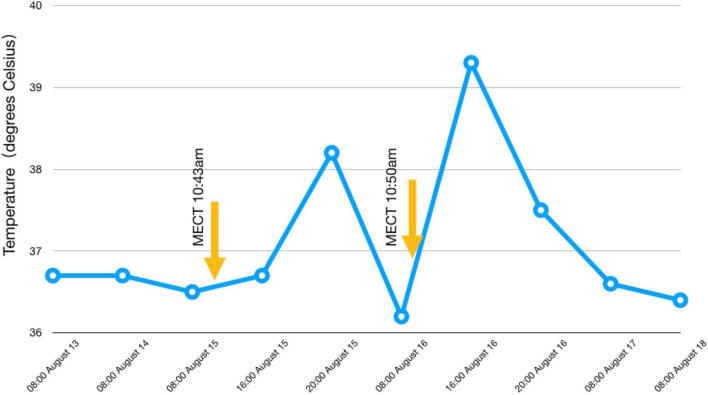
Temperature changes before and after MECT. We can see that the patient’s body temperature increased significantly after MECT.

**FIGURE 3:**
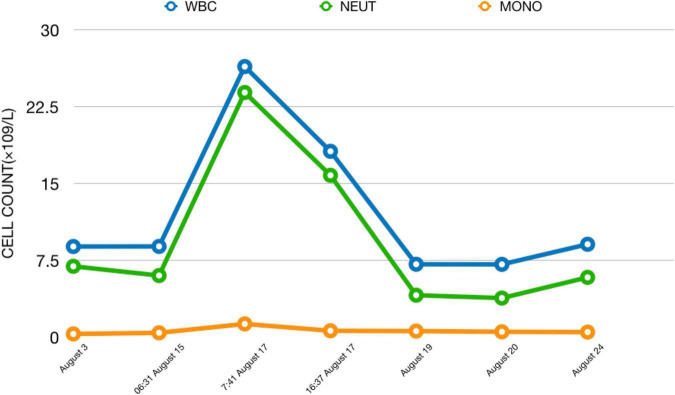
Changes in cell count from admission to discharge. We can see the huge changes in cell count in a short time after this patient received MECT.

On August 16, 2018, we arranged a second modified electrotherapy for the patient. We selected the same MECT parameters as the first time, and the electrotherapy process was smooth, but after returning to the ward, we found that the patient’s temperature gradually increased, up to 39.3 degrees Celsius, accompanied by slight limb vibration lasted nearly 20 min. Even if the patient was conscious and had no other discomfort, we arranged laboratory and imaging examinations while physical cooling. The laboratory used Sysmex’s five category automatic blood analyzer, model xn9000, and the combined detection method of electrical impedance and RF conductivity was used for detection. The examination results showed that the leukocyte count of the patient was 26.40 × 10^9^/L, the absolute value of neutrophils was 23.87 × 10^9^/L, the absolute value of monocytes was 1.29 × 10^9^/L, and inflammation-related factors (procalcitonin, C-reactive protein, interleukin-6), liver and kidney function, aerobic and anaerobic blood culture, urination routine and coagulation function were normal. We also arranged emergency chest computed tomography (CT) and EEG examinations to rule out the possibility of pulmonary infection and epilepsy, and all the results were normal. Then, we invited the infectious disease department, neurology department and hematology department to consult. Only the hematology department recommended hydration treatment to reduce the white blood cell count. The department of infection and neurology did not consider the existence of infection or epilepsy, but even the hematology department cannot give a specific explanation for the sudden increase in white blood cells. Therefore, we added 1,000 ml physiological saline to the patient through the vein and asked the patient’s families to help her drink 1,000 ∼ 1,500 ml of water. In the afternoon of that day, we reexamined the laboratory indexes. The examination results showed that the leukocyte count of the patient was 18.13 × 10^9^/L, the absolute value of neutrophils was 15.79 × 10^9^/L, and the absolute value of monocytes was 0.62 × 10^9^/L. The patient’s body temperature also returned to normal on the same day (Figure 2). The next day, we checked the patient’s blood cell count again, and the test results were normal. The WBC count of the patient was 7.11 × 10^9^/L, the absolute value of neutrophils was 4.09 × 10^9^/L, and the absolute value of monocytes was 0.59 × 10^9^/L. Although the patient felt better in mental and emotional state after MECT than before, we still suspended electrotherapy after that, adjusted the treatment scheme to topiramate capsule 25 mg twice a day, risperidone 3 mg twice a day, benzhexol hydrochloride tablets 2 mg twice a day, and gradually added clozapine tablets. Before discharge, the patient began to agree that she was not tracked, isolated and revealed, at this time, the clozapine dose was 225 mg/d. We conducted laboratory tests on August 20 and August 24, 2018, and the test results remained normal (Figure 3). The patient’s family was unable to take care of the patient in the hospital for a long time because of their work, at their request, we discharged the patient on August 30, 2018. At discharge, we increased the dose of clozapine tablets to 250 mg/d and revised the patient’s diagnosis to PPD. The patient was followed up in the clinic, but the treatment effect was not ideal. On October 11, 2019, the patient was hospitalized again because of aggravation of the symptoms and additional suicidal thoughts, medication was used but MECT was not performed after admission, however, the reason why MECT was not used again cannot be found in the patient’s medical records.

## Clinical Discussion

The ICD-10 also defines PPD as suspicion, excessive attention to oneself, hostility, etc. (1), PPD individuals are characterized by negative emotionality, hypervigilance, cognitive rigidity and an aggressive, hostile disposition (7). According to data from a US study, the number of PPD ranks second among personality disorders (8), but there have been no clinical trials specific to PPD. A systematized, self-psychological model emerged in the 1990s and posited that paranoid delusions arise when an individual is unable to tolerate the discrepancy between an implicit, negative view of the self and a conflicting, idealized positive self-concept. In such individuals, blame must be externalized to another person in the form of paranoid delusions (9). It can be seen that the revised diagnosis of PPD in this case after admission meets the standard.

For the treatment of PPD, previous studies have shown that it may be beneficial to slow down the treatment to “wait” for an unstable emotional response (10), and in this case, we also followed this principle and continued to observe the patient’s changes in the 13 days prior to MECT and slowly increased the dose of the drug, so the treatment method is also reasonable.

After MECT, the patient had a short-term fever and slight trembling of limbs, which resulted in an increase in leukocytes, neutrophils and monocytes. Antigenic and inflammatory stimuli, as well as psychological and physical stressors were involved in the process of leukocytosis (11), so the normal results of PCT, CRP, IL-6, blood culture, urination routine, chest CT and EEG helped us rule out the possibility of infection and epilepsy after MECT basically.

The patient used clozapine tablets, risperidone and benzhexol hydrochloride tablets before receiving MECT and used propranolol hydrochloride, topiramate capsules in a short time. In previous reports, there have been medical records and mechanisms of leukocytosis caused by clozapine (12); however, in this case, we do not consider that the leukocytosis of the patient is related to clozapine because we have used clozapine tablets for 13 days before receiving MECT. During drug treatment and before MECT, there was no hint of leukocytosis in the laboratory examination. Risperidone can cause a decrease in the number of leukocytes, which is inconsistent with this case. Benhexol hydrochloride, propranolol hydrochloride and topiramate capsule have no reports related to leukocytosis; moreover, the medication time cannot be related to leukocytosis, so none of these can lead to the WBC count increasing nearly three times in a short time and then returning to normal in a short time after MECT. More importantly, during the whole process of hospitalization, we did not stop medical treatment, and there was no increase in leukocytes until the patient was discharged from the hospital, which also proved that the leukocytosis had nothing to do with those drugs, so we had to consider that the patient’s fever and leukocytosis were related to MECT, even if previous studies believed that there were no significant differences in WBC counts before and after MECT (13).

We also considered whether anesthetics were the factors leading to fever and leukocytosis. Penehyclidine hydrochloride, succinylcholine chloride and propofol were used in both MECTs. Susuinylcholine chloride may cause malignant hyperthermia, and it mostly occurs when combined with halothane. Propofol may also cause fever. In this case, although the patient’s temperature does not meet the diagnostic criteria of malignant hyperthermia, whether her fever was related to anesthetics or which kind of anesthetics still needs further research and observation. With regard to leukocytosis, there has not been any relevant report on leukocytosis caused by the above three drugs, and the occurrence time and duration of leukocytosis were not consistent with the pharmacokinetic characteristics of anesthetics, so we will not consider that leukocytosis in patients is related to anesthetics.

## Pathogenesis Discussion

Therefore, what is the possible mechanism of transient leukocytosis and fever caused by MECT? Because of the lack of timely examination, we can only infer it. The regulation of the hematopoietic system is related to cytokines, catecholamines, neuropeptides and hormones (11), and the physiological stress response can also result in increased plasma concentrations of stress hormones such as catecholamines and glucocorticoids. This is accompanied by increases in circulating numbers of granulocytes, monocytes, and natural killer cells (14); beyond that, when the human body is in a stringent state, it will release adrenaline, and the release of adrenaline can trigger neutrophils to enter the circulating pool from the marginal pool and then rapidly increase the neutrophil count in peripheral blood within 5 ∼ 10 min (15), which can reasonably explain the large increase in leukocytes and neutrophils in this case. Does catecholamines and glucocorticoids rise in patients with MECT? This was not involved in the current study, and the relevant hormone levels were not measured in time in this case. Previous studies have conducted experiments on the changes in plasma prolactin (PRL) and thyrotropin (TSH) after ECT and showed that a brief, but pronounced, PRL increase together with a small, but consistent, TSH release after ECT (16). Meanwhile, there is evidence that ECT is similar to antidepressants, which can increase the level of norepinephrine and the sensitivity of α1 receptors and serotonin agonists but decrease the sensitivity of presynaptic α2 receptors to increase the binding of 5-HT receptors in the cerebral cortex (17).

Then, we can also boldly speculate that, for some patients, even MECT can cause a stress response in the body, resulting in a rapid increase in adrenocorticotropic hormone (ACTH), and the secretion of adrenaline and noradrenaline causes leukocytosis and even some unexpected symptoms, even sudden death after MECT treatment (18).

To the best of our knowledge, the basic principle of MECT is to induce therapeutic seizures; at present, there is no relevant report on leukocytosis caused by epilepsy. We mentioned that MECT can lead to hormonal changes, resulting in an increase in leukocytes. In fact, epilepsy can also lead to abnormal hormone secretion (19, 20), and hormones can also affect seizures (21). However, previous studies have focused more on the relationship between sex hormones and epilepsy and less on other hormones. First, we do not know whether the patient in this case had abnormal hormone secretion during MECT. In addition, if the abnormal secretion of one or several hormones was indeed caused by MECT, was the abnormal secretion of these hormones caused by current stimulation or by therapeutic seizures? This is the second problem that needs more research and observation. Finally, if it causes abnormal secretion of hormones, whether these hormones are related to leukocytosis is the third problem we need to determine. Therefore, in this case, the cause of leukocytosis is difficult to identify immediately, but in the future, we can find the cause by measuring the relevant hormones in patients receiving MECT and patients with epilepsy.

## Conclusion

In this case, the patient was a young woman. After ineffective antipsychotic treatment, MECT was used. Slight and transient limb vibration occurred during and after MECT, and fever and leukocytosis were found after MECT. We ruled out the possibility of infection and epilepsy and used intravenous rehydration to restore the number of leukocytes to normal, and the results showed that it was effective. This case suggests that while advocating residual treatment approaches in patients with PPD, we should be vigilant against the occurrence of leukocytosis before, during and after MECT, as well as other unreported conditions. At the same time, even as a psychiatrist, we should timely identify and find the causes of unexpected adverse events and know how to deal with emergencies such as leukocytosis, which inevitably requires psychiatrists to have a more comprehensive ability, not limited to psychiatric treatment.

## Data Availability Statement

The original contributions presented in the study are included in the article/supplementary material, further inquiries can be directed to the corresponding author.

## Author Contributions

XM and SL conceptualized the treatment and wrote the first draft of the manuscript. JX, PL, and YL edited the manuscript. YZ, YS, SC, and ZW conducted the literature search and summary. All authors contributed to the article and approved the submitted version.

## Conflict of Interest

The authors declare that the research was conducted in the absence of any commercial or financial relationships that could be construed as a potential conflict of interest.

## Publisher’s Note

All claims expressed in this article are solely those of the authors and do not necessarily represent those of their affiliated organizations, or those of the publisher, the editors and the reviewers. Any product that may be evaluated in this article, or claim that may be made by its manufacturer, is not guaranteed or endorsed by the publisher.
